# Construction of Marker-Free Genetically Modified Maize Using a Heat-Inducible Auto-Excision Vector

**DOI:** 10.3390/genes10050374

**Published:** 2019-05-17

**Authors:** Dengxiang Du, Ruchang Jin, Jinjie Guo, Fangdong Zhang

**Affiliations:** National Key Laboratory of Crop Genetic Improvement and College of Plant Science and Technology, Huazhong Agricultural University, Wuhan 430070, China; ddx@mail.hzau.edu.cn (D.D.); jrc@webmail.hzau.edu.cn (R.J.); GJJ@webmail.hzau.edu.cn (J.G.)

**Keywords:** marker-free, transgenic maize, *egfp*, heat-shock, recombination, *cre/lox*

## Abstract

Gene modification is a promising tool for plant breeding, and gradual application from the laboratory to the field. Selectable marker genes (SMG) are required in the transformation process to simplify the identification of transgenic plants; however, it is more desirable to obtain transgenic plants without selection markers. Transgene integration mediated by site-specific recombination (SSR) systems into the dedicated genomic sites has been demonstrated in a few different plant species. Here, we present an auto-elimination vector system that uses a heat-inducible *Cre* to eliminate the selectable marker from transgenic maize, without the need for repeated transformation or sexual crossing. The vector combines an inducible site-specific recombinase (*hsp70::Cre*) that allows for the precise elimination of the selectable marker gene *egfp* upon heating. This marker gene is used for the initial positive selection of transgenic tissue. The *egfp* also functions as a visual marker to demonstrate the effectiveness of the heat-inducible *Cre*. A second marker gene for anthocyanin pigmentation (*Rsc*) is located outside of the region eliminated by *Cre* and is used for the identification of transgenic offspring in future generations. Using the heat-inducible auto-excision vector, marker-free transgenic maize plants were obtained in a precisely controlled genetic modification process. Genetic and molecular analyses indicated that the inducible auto-excision system was tightly controlled, with highly efficient DNA excision, and provided a highly reliable method to generate marker-free transgenic maize.

## 1. Introduction

Genetic engineering techniques generally involve the addition of gene (single or multiple) integrated into a plant leading to the modification of its genome [1]. Transgene technique can be used to alter the expression patterns of individual gene in a more precise and predictable manner than conventional breeding [2], and has progressed from being a basic laboratory research technique to become the sole provider of new plant varieties worldwide over the last two decades [3]. There is much controversy over genetically modified (GM) crop: the uncertainty of their safety is the main concern of governments, non-governmental organizations, and the general public [4]. A common concern is the marker genes used for selecting transformed plants, asserting that the food produced from GM plants could be harmful to human health due to potential toxicity or allergenicity [5].

Selectable marker genes are essential to plant genetic engineering and used in nearly all transformation procedures to simplify the identification of transgenic plants. Selectable markers, such as hygromycin, kanamycin, or glyphosate, differentiate transformed cells pass resistance toward antibiotics or herbicides to the transformed cells, whereas untransformed cells and tissues are killed by treatment with lethal compounds [6]. These systems not only allow relatively straightforward identification and selection of stably incorporated plants [7], but also are used to follow the inheritance of foreign gene in the segregating generation [8]. Selectable marker genes are required for obtaining transgenic plants, but serve undesirable to be retained in transgenic plants because of their possible toxicity or allergenicity to humans and their unpredictable hazards to the environment [9]. Moreover, the continued presence can pose technological problems because it precludes additional transformation of constructs with the same marker system [10]. Compared with the existing selection marker genes, the selection of novel selection marker genes is an effective method to eliminate their potential risks. According to the mechanism of action of the safety marker developed and the type of agent selected, it can be divided into amino acid metabolism related genes [11,12], hormone related genes [13,14], sugar metabolism related genes [15], stress resistance related genes [16], and fluorescent protein genes [17,18,19]. The use of these selection markers reduces the potential risks of transgenic plants.

**Figure 1:**
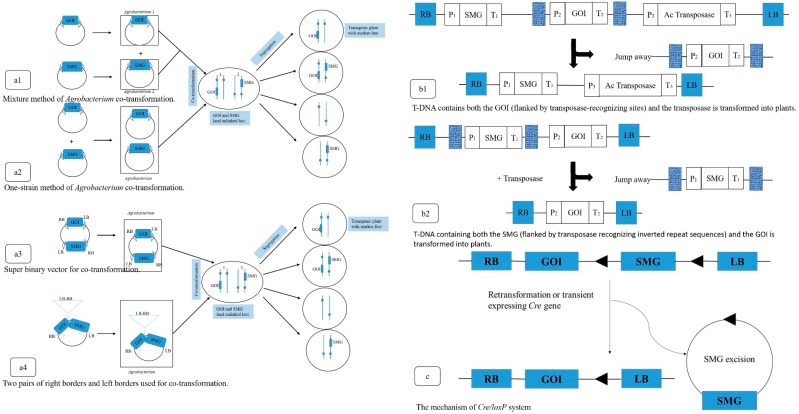
Mechanism of the three approaches for the removal of selectable marker genes from transgenic plants [10]. (**a**) The mechanism of co-transformation to removal of selectable marker genes from transgenic plants. a1, Mixture method of *Agrobacterium* co-transformation. a2, One-strain method of *Agrobacterium* co-transformation. a3, Super binary vector for co-transformation. a4, Two pairs of right borders and left borders used for co-transformation; (**b**) The mechanism of transposon-mediated transgene reintegration to removal of selectable marker genes from transgenic plants. b1, T-DNA contains both the GOI (flanked by transposase-recognizing sites) and the transposase is transformed into plants. b2, T-DNA containing both the SMG (flanked by transposase recognizing inverted repeat sequences) and the GOI is transformed into plants; (**c**) The mechanism of site-specific recombination (SSR) to removal of selectable marker genes from transgenic plants.

The removal of marker genes would likely hasten public acceptance of transgenic crops in a sense [20,21,22], because the production of marker-free transgenic crops is crucial to avoid these potential risks and promote commercial deployment of GM plants [23,24,25]. Several approaches have been developed for the removal of selectable marker genes from transgenic plants [26,27,28,29]. In the first approach, the selectable marker genes and the gene of interest are introduced at different loci of the plant genome by co-transformation (the mechanism is shown in Figure 1(a1–a4), after which the selectable marker gene is segregated out by crossing sexually [30]. The second method entails the elimination of selectable markers by transposition [31,32]. To eliminate the selectable marker using transposon-mediated transgene reintegration is an advantageous strategy for marker gene removal (the mechanism is shown in Figure 1(b1,b2), because it allows intact transgene insertion with defined boundaries and requires only a few primary transformants [33]. The third approach used for the removal of selectable marker genes is by intrachromosomal site-specific recombination (the mechanism is shown in Figure 1c) [34,35,36]. Site-specific recombination (SSR) systems carryout recombination between specific DNA sequences without addition or deletion of nucleotides [37,38,39,40]. There are well-known recombination systems, including *Cre/lox* from bacteriophage P1 [3,41], *Flp/frt* from *Saccharomyces cerevisiae* [42,43], R/RS from *Zygosaccharomyces rouxii* [44], and *Gin/gix* from bacteriophage [45] to remove the marker gene [46].These precisions are highly desirable in plant genetic engineering as they minimize unintended mutations introduced by the transformation process. Marker excision combined with site-specific gene integration requires the use of two SSR systems [13,47], and for a long time, only *Cre/lox* system was popularly used [48].

These simple SSR systems consist of two basic components: a recombination enzyme and small DNA recognition sites, enabling a variety of applications such as site-specific integration, copy number reduction, and marker gene removal [49,50,51,52]. A number of strategies can be used with *Cre/lox* to remove marker genes from transgenic plants and each strategy differs in how *Cre* function is delivered [41,53]. In all strategies, directly repeated *lox* sites flank the marker gene and excision occurs when *Cre* is active. In one strategy, similar to the co-transformation of the transposon system, transgenic plants that carry a marker gene flanked by *lox* sites can be retransformed with a *Cre*-expressing plasmid, and the *Cre* gene segregates in the next generation [47,54,55,56]. Alternatively, marker gene flanked by *lox* sites and *Cre*-expressing construction were on two plasmids, whereby *lox* plants can be crossed with plants that express *Cre* (herein referred to as the “crossing strategy”); in this case, marker excision occurs in the F_1_ progeny followed by the loss of the *Cre* gene by genetic segregation in the F_2_ generation [57,58,59,60].

The third strategy is referred to as the “induction-excision strategy”; in this strategy, *Cre* and selective marker genes are built between two *lox* sites directly, through induction type promoter or driven by cell-specific promoter for *Cre* recombinant enzyme gene expression, to remove selection marker gene to solve the simple delete operation [38,47]. Gleave et al. [61] successfully deleted the marker genes by expressing the *Cre* recombinase in transgenic tobacco, thus avoiding the process of retransformation and hybridization. Based on this principle, Hoff et al. [62] constructed a *pCrox* vector system: the thermal promoter GSP81-1Cre induced the *Cre* recombinase gene expression, and eliminated the kanamycin resistance gene which was inserted between the two *loxP* sites in transgenic *Arabidopsis thaliana*. An important improvement of this early technique was reported by Zuo et al. [63] using a chemically inducible artificial transcription factor for indirect transcriptional regulation of *Cre*-recombinase gene expression. Thus, the recombinase gene and the lox recombination sites could coexist without leading to premature recombination. Following the selection of transgenic tissue, chemical induction of the recombinase gene produced the desired excision events. Heat-inducible promoters are the widely used and studied promoters for driving site-specific recombinases [42,56,64,65,66,67].

In maize, co-transformation strategies [68,69,70,71] and transposon-induced recombination [72] have been reported to remove the selection marker genes. The *Cre/lox* system has also attracted the attention of maize researchers. In 2003, Zhang et al. [3] first reported the use of site-specific recombination to eliminate the selection marker gene in the transgenic maize. The researchers used the strategy to transfer the *Cre*-recombinase gene and the selection marker gene with lox sequence into two maize transformed lines. The *Cre/lox* system activated in the F_1_ hybridization and marker-free transgenic maize were separated in backcross material. Vega et al. [73] constructed a BIBAC vector system to adapt for maize transformation with a chemically induced marker elimination system, and avoided the complex operation of separating hybrid offspring. Based on the previous studies, site-specific recombinant system *Cre/lox* that was used to eliminate the selection markers from transgenic maize should solve two drawbacks: the difficulty in establishing a control elimination system and the formation of genetic chimeras due to incomplete DNA excision. For this purpose, the following works were carried out in this study: (i) We constructed a heat-inducible auto-excision vector using a heat-shock-responsive *HSP70* promoter [74,75,76,77]; compared to previously reported systems, this new system is tightly controlled and the induced DNA excision is highly efficient. (ii) We used green fluorescent protein gene (*egfp*) as selection marker to screen transgenic callus. Compared with the existing herbicide resistance markers, this marker has the advantages of convenient detection and no harm to living cells in the screening process. In addition, the visible fluorescence phenotype can effectively identify the elimination of selection marker genes [18]. In the present study, marker-free transgenic maize plants were obtained via recombination-programmed auto-excision without any extra handling borrowed from the traditional transformation process with a few modifications by the vector.

## 2. Materials and Methods

### 2.1. Plant Material

Hi-II hybrid maize (A188 × B73 origin) was used as the plant material and was cultivated on an experimental farm at Huazhong Agricultural University in Wuhan, China. Following self-pollination at approximately 10–14 days, the ears were harvested when the immature embryos were 1.0–2.0 mm in length. To sterilize their surface, the ears were incubated in 70% ethanol for 5 min and then in 2.4% sodium hypochlorite supplemented with 0.1% Tween for 20 min, before being washed five times in sterile distilled water for 5 min every time. Immature embryos of normal and healthy morphology were isolated with a lancet and collected in a 2 mL tube containing 1 mL of sterile distilled water. A type II callus, which is clumps of nonpolar cells with bright color, loose structure, granular shape, dry surface, and rapid growth, was generated after 60–90 days in the induction medium.

### 2.2. Construction of Automatic Elimination Vector

**Figure 2:**
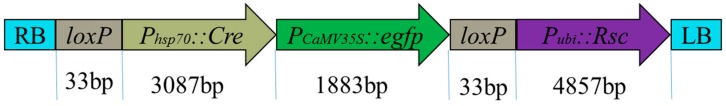
Transfer DNA (T-DNA) organization of the binary plasmid. LB is left border; RB is right border. loxP is the recombination-site (*lox*); *P_hsp70_::Cre* is the *Cre* gene carrying a nuclear localization signal with the heat-shock-responsive promoter *Hsp70*; *P_CaMV35S_::egfp* is *egfp* gene as visual marker for selection driven by the CaMV35S (35S) promoter; *P_ubi_::Rsc* is a reporter gene anthocyanin pigmentation (*Rsc*), controlled by the maize polyubiquitin gene (*ubi*) promoter.

The goal of this strategy is to obtain transformation and marker auto-excision in a single process using an induced expression site-specific recombination system. We constructed a vector using the skeleton of *pEGAD*, which is schematically represented in Figure 2. The *egfp* gene driven by the CaMV35S promoter functioned as a transformation selectable marker, constructed to assemble the DNA fragments of the selection element [78,79]. The *egfp* carried by the vector was used for two purposes. First, green fluorescent protein expression functioned as a visual selection signal that allows the direct visualization of transformed cells. Second, the loss of green fluorescence in the heat-shock-treated calli served as a counter-selectable marker that effectively eliminates the recombination element and selection markers. To remove the selection markers from the resulting transfer DNA (T-DNA), we employed the *Cre/lox* system [53]. The bacteriophage *Cre* recombinase specifically recognizes both *lox* sites in transformation cells. The recombinase *Cre* gene carrying a nuclear localization signal and the heat-shock promoter Hsp70 from maize [75,76,77], which is used to control its expression, was positioned between a *lox* site and the selection element. The whole element was flanked by two *lox* sites. Further, the heat-inducible *cre* gene located within the two *lox* sites can be removed by inducing *Cre* activity at any time after selection of the transgenic clones [80]. To test the efficacy of the vector system described above, we cloned the polyubiquitin gene (*ubi*) promoter [81] and controlled a maize anthocyanin pigmentation (*Rsc*) gene [82] into the construct (*pHZM1N-Rsc*) between the *lox* site and the left border (LB).

The vector was compounded by Make Research Easy (Nanjing, China) and was transferred into *Agrobacterium tumefaciens* strain EHA105 by electroporation. The bacteria were precultivated for 2–3 days on LB solid medium with 100 mg/L kanamycin at 28 °C in the dark [79]. On the day of transformation, *Agrobacterium* colonies were collected from the plate with a spatula, resuspended in Infection medium with 100 μM acetosyringone (Table 1), and incubated for 2–3 h at 28 °C and 200 rpm [79]. Cell density, measured as OD600, was adjusted prior to callus infection (see below).

### 2.3. Media

Media preparations and related procedures were performed as described previously [79], with the specific compositions listed in Table 1.

### 2.4. Plant Transformation by Agrobacterium Transgenic System on Calli

Plant transformation followed an established standard protocol with some modifications [79]. Embryonic calli were collected in 10 mL microcentrifuge tubes containing liquid infection medium with 100 μM acetosyringone and were allowed to sit for 20 min. The solution was drawn off, the calli were then infected with 5 mL of *Agrobacterium* suspension containing the *pHZM1N-Rsc* binary vector, followed by brief vortexing for 5 min at 28 °C. The *Agrobacterium* liquid was drawn off and the infected calli were transferred onto the solidified co-cultivation medium, and were tiled on the medium (Table 1). The plate was sealed with Parafilm and incubated in the dark at 19 °C for 3 days. After co-cultivation, the calli were rinsed 4 times with sterile distilled water followed by 2 times (5 min) with sterile water containing 100 mg L^−1^ carbenicillin (ICN, Costa Mesa, CA, USA) and were blotted dry on a sterile filter paper. They were then transferred onto the resting medium (Table 1) containing 100 mg L^−1^ carbenicillin (ICN, Costa Mesa, CA, USA) and incubated at a temperature of 28 ± 2 °C for 7 days in the dark.

### 2.5. GFP Fluorescence Assay

After 7 days restoration of culture, the instantaneous conversion efficiency was calculated by observing the green fluorescence performance on a DR-46B Dark Reader transilluminator (Clare Chemical Research, Dolores, CO, USA) and a digital camera (a550; Sony, Tokyo, Japan) with a GFP filter. Then, the calli were transferred to selection medium (Table 1) to selection culture in the dark at 28 ± 2 °C. Two weeks after the 1st round of selection, tissues were transferred to fresh selection medium and sub-cultured at 2–3 weeks intervals. After the third round of selection, the conversion efficiency was calculated by observing the green fluorescence performance on the DR-46B Dark Reader transilluminator; the statistical method showed the same instantaneous conversion efficiency as calculated by the experiment [32,79].

### 2.6. Heat-Shock Induction

The selected GFP-positive calli were broken into small pieces (1 × 1 mm) and transformed onto selection media in glass plates after three rounds of GFP fluorescence performance selection. The glass plates were transferred to a 42 °C incubator for 2 h in the dark to induce *Cre* activity as described [66,83]. The calli were then transferred onto new resting medium containing 100 mg/L carbenicillin (ICN, Costa Mesa, CA, USA) and incubated at 28 ± 2 °C in the dark for 7 days. After 3 weeks of subculture on selection medium at 28 ± 2 °C in the dark, the loss of GFP expression was used to examine the auto-excision efficiency of the *cre* recombinase; GFP-negative callus blocks from calli were subcultured from each independent experiment, and the calli with green fluorescence expression were transferred to a 42 °C incubator and kept for 2 h in the dark for heat-shock treatment for the second time. After two rounds of selection, the green fluorescence phenotypic characterization in maize calli were detected with a fluorescent protein macro detector set (Nikon, Tokyo, Japan); the GFP-negative callus blocks from calli were subcultured and redifferentiated. Regenerative green tissues were then transferred to regeneration medium (Table 1) and incubated at 28 ± 2 °C under dim light (10–30 mE m^−2^ s^−1^, 16/8 h day and night photoperiod) for 2–3 weeks. The shoots were transferred to rooting medium (Table 1) and exposed to higher light intensity (50–150 mE m^−2^ s^−1^, 16/8 h day and night photoperiod) for rooting. The plantlets were then transferred to soil [79].

### 2.7. Maize Genomic DNA Extraction

Maize calli and/or leaf tissue was frozen in liquid nitrogen and ground to a fine powder. Genomic DNA was extracted by the CTAB (hexadecyltrimethylammonium bromide) method from the frozen tissue. Callus or seedling leaves broken by physical means was incubated in a water bath at 65 °C for 40 min in a tube containing 500 µL CTAB buffer, and then centrifuged at 12,000 rpm for 10 min. The supernatant was transferred into a new tube, and an equal volume of isopropanol was added [79]. The solution was gently mixed and then centrifuged at 12,000 rpm for 5 min. After washing with 75% (*v*/*v*) ethanol and drying in air, the DNA pellet was dissolved in 2 mL of distilled water [79].

### 2.8. PCR Amplification of *egfp*, *Rsc* and Residual Sequence (RS) in Transgenic Maize Plants

The PCR monitoring area and probe location are shown in Figure 2b. The selection marker gene *egfp*, the *Rsc* gene, and the residual sequence (RS) after the marker was eliminated were amplified by PCR using the primer pairs P1 (5′-GGACTGGGTGCTCAGGTAGTGG-3′; 5′-CTGGACGGCGACGTAAACGG-3′), P2 (5′-GGGTTTAGGGTTAATGGT-3′; 5′-CACTGGCAAGTTAGCAAT-3′), and P3 (5′-AAACGGAGCATAGAGGATA-3′; 5′-CACTGGCAAGTTAGCAAT-3′), respectively. The expected size of the *egfp*-PCR product was 879 bp, and the expected size of the Rsc-PCR product was 465 bp, which contained part of the sequence of the *ubi* promoter and part of the *Rsc* gene. The size of the selection construct was initially 6836 bp, and it was 1729 bp after marker elimination. The cycling parameter was 94 °C for 5 min; 33 cycles of 94 °C for 30 s, X °C for 30 s, and 72 °C for 2 min; and 72 °C for 5 min. The annealing temperatures (X marks in the parameter) were 55 °C for *egfp*-PCR, 58 °C for Rsc-PCR and 50 °C for residual sequence (RS), separately.

### 2.9. Southern Blot Analysis

Southern-blot hybridization was conducted using the PCR DIG Probe Synthesis Kit and the DIG High Prime DNA Labeling and Detection Starter Kit II (Roche, Basel, Switzerland) according to the manufacturer’s instructions. Genomic DNA of the transgenic plants was extracted by the CTAB method [84] from GFP-positive callus. For Southern blot analysis, 25 μm of maize genomic DNA from each sample was digested to completion with 10 units of *Asc*I, fractionated on a 1% (*w*/*v*) agarose gel, and transferred onto an Amersham Hybond N^++^ membrane (GE Healthcare, Chicago, IL, USA) according to the instructions [85]. Probe 1 (Rsc: 465 bp, 5′-GGGTTTAGGGTTAATGGT-3′ and 5′-CACTGGCAAGTTAGCAAT-3′) was synthesized using the PCR DIG Probe Synthesis Kit according to the manufacturer’s instructions. DNA membranes were hybridized with the probes in DIG Easy Hyb (Roche) solution at 42 °C. After hybridization, the membranes were washed at 65 °C two times with 2 × SSC and 0.1% (*w*/*v*) SDS for 10 min and two times with 0.1 × SSC and 0.1% (*w*/*v*) SDS for 15 min [36]. Probe hybridization signal was examined by digoxigenin chemiluminescence detection. Membranes were stripped afterward by washing at 37 °C two times with 0.2 N NaOH and 0.1% (*w*/*v*) SDS for 30 min and one time with 2 × SSC for 5 min. Further hybridization was performed using the *Asc*I marker (New England Biolabs, Ipswich, MA, USA) that was labeled using the DIG High Prime DNA Labeling Kit.

## 3. Results

### 3.1. Determination of Transformed Callus

**Figure 3:**
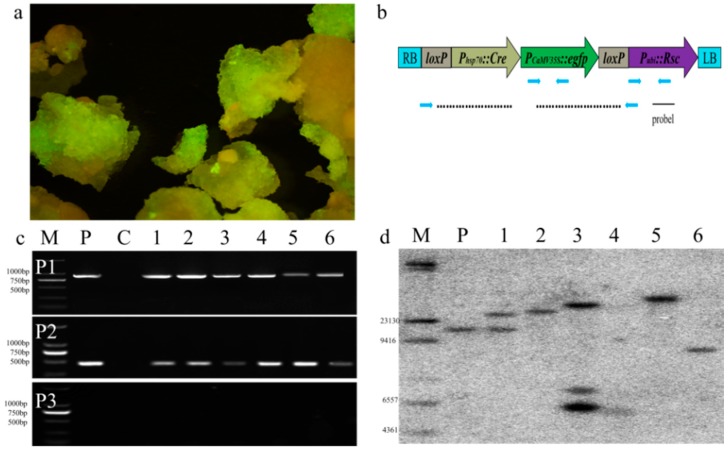
Determination of transformed callus. (**a**) The GFP phenotype of the transformation-positive calli on the DR-46B Dark Reader transilluminator at 3 weeks after infection, the non-fluorescing parts due to the callus being untransformed; (**b**) The PCR monitoring area and probe location in T-DNA organization of pHZM1-Rsc. Specific primer sites frame the T-DNA. P1 detected the selection marker *egfp*, P2 detected the regulator gene (*Rsc*), and P3 was used to detect the RS after the markers were eliminated. Probe 1 is on the gene *Rsc*, beside the restriction of endonuclease site *Asc* I, which is used as a marker for the southern blotting probe. (**c**) PCR analysis of the transformed maize calli. DNA was extracted from six independent transgenic GFP^+^ maize calli; specific primer pairs P1, P2, and P3 were used to detect the selection marker gene *egfp*, the regulator gene (*Rsc*), and the RS, respectively. M, BM2000 DNA marker; P, positive control P was used as the plasmid DNA; C, negative control; lanes no. 1–6, DNA from independent transgenic calli. (**d**) Southern blot analysis of the transferred gene in independent transgenic experiments. M, HindIII DNA Marker; P, positive control P was used as the plasmid DNA; lanes no 1–6, DNA from independent transgenic calli.

High quality embryogenic calli of Hi-ΙΙ maize were chosen for highly efficient maize transformation achieved using the *Agrobacterium*–mediated methods shown by Du et al. [79]. Three weeks after an infection, we obtained 200 transgenic calli when GFP fluorescence phenotypic (Figure 3a) assayed on the DR-46B Dark Reader transilluminator. The GFP phenotype was because of the transformation-positive calli, while the non-fluorescing parts were due to the callus being untransformed. The transformation efficiency, defined as the weight ratio of the calli with GFP activity to the total weight of co-cultivated calli that produced secondary calli, was calculated to be approximately 16.7% at three weeks of culture selection from the co-cultivated calli.

Six transgenic events, based on the fluorescence assays, were chosen to analysis by PCR and Southern blotting. The PCR monitoring area and probe location are shown in Figure 3b and the results are shown in Figure 3c: P1 and P2 produced the expected product sizes; the PCR bands were 879 bp and 465 bp, respectively, for all six events, but no band was amplified from any of the seven calli by P3 because the product is too large for the given PCR conditions. An additional independent confirmation was obtained by Southern blot analysis. Genomic DNA from each event was digested and hybridized with probes specific to the polyubiquitin gene (*ubi*) promoter and the maize reporter gene (*Rsc*) linked sequence, as revealed for all six events in Figure 3d. Most transgenic plants contained a single band of the transgene. No. 2, No. 5, and No. 6 had a single fragment; however, the sizes of the copies were different. No. 2 and No. 5 had larger fragments compared to the positive control, while No. 6 with had a smaller band. No. 1 had two bands, and No. 3 had three bands. No. 4 perhaps had one smaller band at the similar size with one band in No. 3.

### 3.2. Induction of Marker Excision and Molecular Analysis of Calli

**Figure 4:**
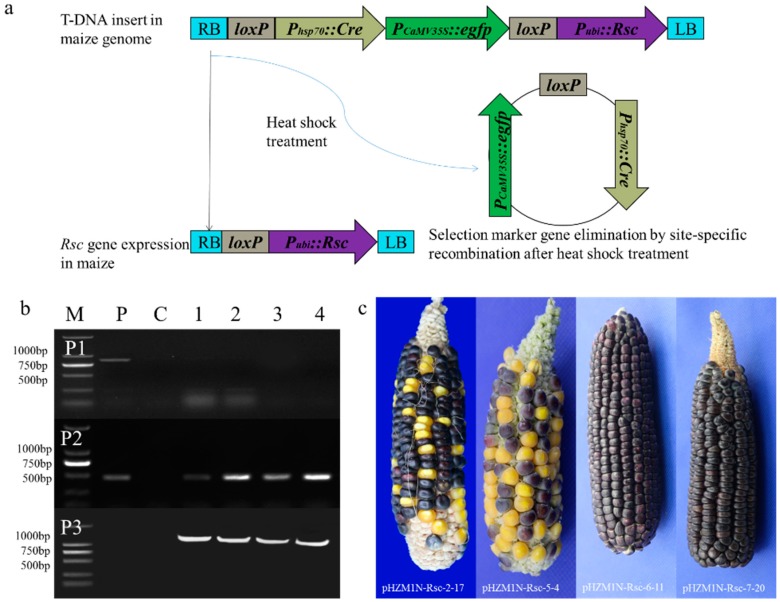
Induction of marker excision and molecular analysis of regenerated plants. (**a**) Design of DNA constructs and molecular strategy of marker-free site-specific gene integration. (**b**) PCR analysis of the transformed maize lines. Specific primer pairs P1, P2, and P3 to detect the selection marker gene *egfp*, the regulator gene (*Rsc*), and the residual sequence (RS), respectively. M, BM2000 DNA marker; P, positive control P was used as the plasmid DNA; C, negative control; lanes no. 1–4, DNA from independent transgenic transformants plants; (**c**) *Rsc* gene expression in genetically modified materials in T_1_ generation. Wild type ears appeared with yellow and white kernels, while the transformation ears with the *Rsc* gene expression phenotype showed a lot of purple kernels.

The transformed calli containing a single copy were chosen for marker excision by heat-shock treatment; the loss of GFP expression was used to examine the auto-excision efficiency of the *cre* recombinase (schematic diagram shown in Figure 4a). After the first heat-shock treatment, the calli were transferred onto the resting medium for 7 days and then to subcultured on selection medium at 28 ± 2 °C in the dark for 3 weeks. The frequency of elimination condition was 21.95%, 6.38%, 19.05%, and 16.28%, by the mean percentage of GFP-negative callus blocks to calli subcultured from each independent experiment (Table 2). The selection marker gene had not been successfully removed in the calli in which green fluorescence had not completely disappeared, and those calli were treated for the second round of heat-shock-induction treatment. After two rounds of heat-shock treatment, the calli were transferred onto the resting medium at 28 ± 2 °C in the dark for 7 days. After 3 weeks, subcultured on selection medium at 28 ± 2 °C in the dark, callus blocks without the green fluorescence phenotype were characterized with a fluorescent protein macro detector set (Nikon, Tokyo, Japan) as the GFP-negative callus blocks. In addition, PCR analysis also obtained the product of the anthocyanin pigmentation gene (*Rsc*) with the 465 bp banding, marker gene detection product disappeared at the same time, and primer P3 PCR analysis obtained expectant 1729 bp band after marker elimination (Figure 4b). These results illustrate that the *Rsc* gene remains stable in the callus genome after heat-shock treatment but that the selection marker was eliminated, which is consistent with the effect on GFP expression. Conversely, due to the elimination of the selection marker, the fragment between the LB and the *Rsc* gene greatly shortened. A 1729-bp PCR product was detected with the P3 primers, as expected for excision of the reporter gene. At the same time, the positive control was too large for the given PCR conditions. In summary, PCR analysis demonstrated the efficient excision of both fragments flanking the selection marker and recombinant enzyme construct after heat treatment of the GFP-positive calli derived from four different transformations. Next, the inheritance of the marker-free locus was studied by analyzing the progeny of these plant lines [86].

### 3.3. Regenerated, Molecular Analyzed, and Phenotypes Authenticate of Transgenic Plants

The transformed plants (T_0_) were regenerated from four independent events. Plants were transplanted into soil and transferred to greenhouse, where they grew for 2–3 weeks before harvesting leaves for the isolation of genomic DNA. Selection marker excision was detected in 100% (17/17) of the tested plants, while the transformed anthocyanin pigmentation gene (*Rsc*) remained integrated in the transgenic plants (Table 3). The maize seeds of each T_0_ line were harvested to examine the expression of the regulator gene to distinguish transformed from non-transformed seeds (Figure 4c).

Transformed seeds were randomly selected from each of the cultivar combinations for sowing. A total of 113 regulator-positive T_1_ plants were used for DNA analysis using regulator-specific primers (P2) and individual plants were further analyzed using PCR to amplify the *gfp* (P1) and the RS of the marker-eliminated sequence (P3). The parents of all the selected marker-free T_1_ plants showed the *rsc*^+^*gfp*^−^RS^+^, as shown by PCR results. The segregation ratio of purple seeds to yellow seeds in each T_1_ line was investigated using the χ^2^ test by the percentage count of purple and yellow grains on the ear (Table 4). Fifteen (88%) lines segregated with a 3:1 Mendelian pattern of inheritance, and the other two (12%) lines segregated with non-Mendelian ratios of distortion toward non-regulator seeds. The progeny of each T_0_ plant showed a complete marker excision, and a high percentage of T_1_ progeny from each T_0_ line is expected to contain a marker-free locus, which was confirmed by PCR on the excision footprint (RS).

## 4. Discussion

The transformation of most crop species has been rapidly achieved using various techniques, but it’s always relatively inefficient no matter which is used [87]. Successful genetic transformation requires not only efficient gene delivery but also an efficient selection system [88] that actively separates the transformed cells that are capable of regenerating plants from non-transformed cells. Currently, a gene with resistance to antibiotics or herbicides is usually selected in maize as the screening marker of transgenic genes. Selectable markers allow the transformed cells survive to tolerate an otherwise lethal exposure and grow into plants, and when the untransformed tissues are killed [89]. Without them, the creation of transgenic crops is not feasible on purely economic and practical terms [90]. There is concern that antibiotic resistance genes in genetic modification crops has chance to flow of into the genomes of the microorganisms living in the soil, might lead to the development of antibiotic-resistant pathogens [23,91]. A similar concern arises when genes that confer herbicide resistance are present; some people fear that super weed will be created by crosspollination between a genetic modification and wild (weedy) [92].

To avoid the above-mentioned risks, the screening of new relatively safe selection marker genes is an effective method to eliminate the potential risk of screening marker genes [6,9,93]. The use of fluorescent protein genes reduces the potential risks, to produce transgenic crops without any other sequences that are not desired in the final product [94]. Since its introduction in 1994 [95], green fluorescent protein has been utilized successfully as a marker in many species [17,19,74], and provided a more efficient direct visual screening method. This study constructed *egfp* gene instead of the bar gene as a transformation selectable marker. Transgenic positive callus was obtained by green fluorescence phenotype as a visual selection signal that allows the direct visualization of transformed cells. A total of 16.7% of the conversion efficiency was basically obtained from transformed callus. Results show that GFP would be useful for identifying transformed tissue, as we have shown that when GFP is synthesized and resides in the callus it accumulates or fluoresces and the transformed cells can be isolated easily and accurately in living callus. In addition, we found no interference with the subsequent growth of transformed maize calli in the selection stage. Second, the loss of green fluorescence in the heat-shock-treated calli served as a counter-selectable marker that effectively eliminates the recombination element and selection markers.

These new marker genes are regarded as less risky [40,96], the complete elimination of selectable marker gene has always been considered the most elegant way to overcome all the concerns [97]. Site-specific recombinant system is an important system to obtain marker elimination of transgenic plants [45], and *Cre/lox* recombinant system is the most widely used site-specific recombinant system [33]. Retransformation, crossing, and auto-excision are three successful application strategies for using the *Cre/lox* system to remove marker genes from transgenic plants [3]. The goal of our strategy is to obtain transformation and marker auto-excision in a single process using an induced expression of site-specific recombination system. By employing the well-known heat-inducible *Cre/lox* system (based on the use of the maize heat-shock *hsp70* gene promoter described), it was easy to generate marker-free first-generation (T_0_) transgenic plants because sexual crosses are not required for selectable marker gene elimination. These plants obtain marker-free transformation before regeneration, through complete control and detection, and can transmit the target gene expression to the next generation stably, which improves the efficiency of safe transgenic plants and optimizes the operation process [65].

Although this is not the first reported use of *Cre/lox* system for marker-free transgenic maize operations, our strategy and vector system have their own advantages [3]. In Zhang’s reports [3], the researchers transformed the recombinase gene *cre* and selection marker gene with lox sites in maize, separately. Then the *Cre/lox* system activated in the F_1_ hybridization and marker-free transgenic maize were separated in backcross material. We constructed a heat-inducible auto-excision vector using a heat-shock-responsive *Hsp70* promoter [74,75,76,77]; compared to previously reported systems, this new system is tightly controlled and the induced DNA excision is highly efficient. It is well known that the preparation and tracking of samples is time-consuming when screening large numbers of transformed molecules, and even worse, the false positive cells can hardly be identified from the tissue with the elimination of markers [21]. Compared with Vega’s work [73], we used green fluorescent protein gene (*egfp*) as selection marker to screen transgenic callus. Compared with the existing herbicide resistance markers, this marker has the advantages of convenient detection and no harm to living cells in the screening process. In addition, the visible fluorescence phenotype can effectively identify the elimination of selection marker genes [18]. In the present study, marker-free transgenic maize plants were obtained via recombination-programmed auto-excision without any extra handling borrowed from the traditional transformation process with a few modifications by the vector. The same mechanism has been successfully used for making marker-free hybrid aspen [48], potato [64], and rice [65].

## 5. Conclusions

In conclusion, an efficient method to combine the advantages of precise genetic engineering with marker gene excision into a single platform is proposed. The introduction of the visual selection markers *egfp* significantly improves the filtering accuracy and simplifies the operation.

## Figures and Tables

**Table 1:** Composition of media used in *Agrobacterium*-mediated transformations.

Medium	Composition
LB (solid)	Yeast extract 5 g/L, NaCl 10 g/L, peptone 10 g/L, agar 15 g/L, pH 6.8
LB (liquid)	Yeast extract 5 g/L, NaCl 10 g/L, peptone 10 g/L, pH 6.8
Infection	N6 ^1^ 2 g/L, 2,4-D ^1^ 2.0 mg/L, L-proline 0.7 g/L, sucrose 68.4 g/L, D-glucose ^2^ 36 g/L, MES ^1^ 0.5 g/L, myo-inositol 0.1 g/L, As ^1,2^ 200 μM, pH 5.2
Co-cultivation	N6 4 g/L, 2,4-D 2.0 mg/L, L-proline 0.7 g/L, sucrose 30 g/L, MES 0.5 g/L, myo-inositol 0.1 g/L, CuSO_4_^1,2^ 0.05 µM, DTT ^1,2^ 1 M, L-cysteine 0.4 g/L, As 100 μM, agar 8 g/L, pH 5.8
Resting	N6 4 g/L, 2,4-D 2.0 mg/L, L-proline 0.7 g/L, sucrose 30 g/L, MES 0.5 g/L, myo-inositol 0.1 g/L, AgNO_3_^1,2^ 0.85 mg/L, carbenicillin ^1,2^ 0.1 g/L, gelrite 2.5 g/L, pH 5.8
Selection	Resting medium without carbenicillin, pH 5.8
Regeneration	MS ^1^ 4.3 g/L, sucrose 30 g/L, myo-inositol 0.1 g/L, 6-BA 3.5 mg/L, gelrite 3.0 g/L, pH 5.8
Rooting	MS 4.3 g/L, sucrose 25 g/L, NAA 0.5 mg/L, gelrite 2 g/L, pH 5.8

^1^ 6-BA, 6-Benzylaminopurine; N6, Chu medium salt with N6 vitamins; 2,4-D, 2,4-dichlorophenoxyacetic acid; MES, 2-(N-morpholino) ethanesulfonic acid; As, acetosyringone; CuSO_4_, copper sulfate; DTT, dithiothreitol; AgNO_3_, silver nitrate; MS, MS basal salt and vitamins; NAA, 1-Naphthaleneacetic acid. ^2^ Components were filter sterilized.

**Table 2:** GFP fluorescence assays and PCR analysis of the auto-excision marker in calli.

**Cultivar**	**First Heat Shock**			
	**GFP Fluorescence Assays**		**PCR Analysis of Non-Fluorescent Calli**	
	**Positive**	**Negative**	***gfp* Detected (+/−)**	***rsc* Detected (+/−)**
pHZM1N-Rsc-2	9	41	9/41	50/50
pHZM1N-Rsc-5	3	47	3/47	50/50
pHZM1N-Rsc-6	8	42	8/42	50/50
pHZM1N-Rsc-7	7	43	7/43	50/50
**Cultivar**	**Second Heat Shock**			
	**GFP Fluorescence Assays**		**PCR Analysis of Non-Fluorescent Calli**	
	**Positive**	**Negative**	***gfp* Detected (+/−)**	***rsc* Detected (+/−)**
pHZM1N-Rsc-2	0	50	0/50	50/50
pHZM1N-Rsc-5	0	50	0/50	50/50
pHZM1N-Rsc-6	0	50	0/50	50/50
pHZM1N-Rsc-7	0	50	0/50	50/50

**Table 3:** PCR analysis of T_1_ transgenic maize plants.

Cultivar	Plants ^1^	PCR Analysis of T1 Transgenic Plants			
		P1	P2	P3	*rsc*^+^*gfp*^−^RS^+^
pHZM1N-Rsc-2	9	0	9	9	9
pHZM1N-Rsc-5	5	0	5	5	5
pHZM1N-Rsc-6	1	0	1	1	1
pHZM1N-Rsc-7	2	0	2	2	2

^1^ Transformed seeds were randomly selected from each of the cultivar combinations for sowing, and their leaf samples were bulked for DNA purification. The bulked maize DNA was then amplified using primers to select *gfp* (P1), regulator-specific sequences (P2), and the RS resulting from marker-eliminated sequences (P3).

**Table 4:** Segregation ratio analysis of T_1_ kernel.

Cultivar	Plants	Purple Seeds	Yellow Seeds	Segregation Ratio	Consistency with 3:1
pHZM1N-Rsc-2	pHZM1N-Rsc-2-1	307	96	3.20:1	Yes
	pHZM1N-Rsc-2-2	218	75	2.91:1	Yes
	pHZM1N-Rsc-2-3	114	36	3.17:1	Yes
	pHZM1N-Rsc-2-5	222	68	3.26:1	Yes
	pHZM1N-Rsc-2-7	256	80	3.20:1	Yes
	pHZM1N-Rsc-2-8	187	71	2.63:1	No
	pHZM1N-Rsc-2-5	299	113	2.65:1	No
	pHZM1N-Rsc-2-16	196	69	2.84:1	Yes
	pHZM1N-Rsc-2-17	185	51	3.63:1	No
pHZM1N-Rsc-5	pHZM1N-Rsc-5-1	209	71	2.94:1	Yes
	pHZM1N-Rsc-5-2	179	55	3.25:1	Yes
	pHZM1N-Rsc-5-3	315	107	2.94:1	Yes
	pHZM1N-Rsc-5-4	122	41	2.96:1	Yes
	pHZM1N-Rsc-5-5	237	64	3.70:1	Yes
pHZM1N-Rsc-6	pHZM1N-Rsc-6-11	374	0		No
pHZM1N-Rsc-7	pHZM1N-Rsc-7-3	191	67	2.85:1	Yes
	pHZM1N-Rsc-7-20	409	0		No
